# Sampling Techniques on Collecting Fine Carbon Nanotube Fibers for Exposure Assessment

**DOI:** 10.1038/s41598-019-43661-1

**Published:** 2019-05-09

**Authors:** Jared Khattak, Daniel Theisen, Candace S. J. Tsai

**Affiliations:** Department of Environmental & Radiological Health Sciences, College of Veterinary Medicine & Biomedical Sciences, Colorado State University, Fort Collins, 80523 CO USA

**Keywords:** Environmental impact, Health occupations

## Abstract

Carbon nanotube (CNT) sampling using an open-faced 25 mm cassette fiber sampling method and a newly developed direct sampling device was evaluated for the size fractioned analysis of collected airborne CNT fibers to improve the sampling and analytical methods. The open-faced 25 mm cassette fiber sampling method primarily collected large agglomerates, with the majority of collected particles being larger than two micrometer in size. Most of CNT structures collected by the new direct sampling device were individual fibers and clusters smaller than one micrometer with a high particle number concentration discrepancy compared to the open-faced 25 mm cassette method raising the concern of this sampling method to representatively characterize the respirable size fraction of CNT aerosols. This work demonstrates that a specialized technique is needed for collecting small fibers to provide a more representative estimate of exposure. It is recommended that an additional sampler be used to directly collect and analyze small fibers in addition to the widely accepted sampling method which utilizes an open-faced 25 mm cassette.

## Introduction

Fibrous carbon were first identified in the 1890’s during the birth of carbon fiber research^1^. Through the 1950s to 1970s carbon nanotube research experienced a period of rediscovery where a variety of synthesis techniques were developed^2–4^. The synthesis methods were further advanced in early 1990s^5^. Since their inception, CNTs have been increasingly utilized in many industries because of their exceptional physical and chemical properties^6^. In the last decade, CNT exposure has been of growing concern because of their morphological similarities to asbestos^7–10^. CNT toxicity has been demonstrated in a number of animal studies, which suggest they may have a significant impact on pulmonary cells by inducing inflammation, granulomas, and fibrotic reactions^11–15^. Recent studies have shown the potential carcinogenicity of type 7 multi-walled CNTs resulting in 2B carcinogen classification in 2014^16,17^.

CNTs are respirable particles with individual fibers which have been observed to be 4 to 100 nm in diameter and 50 nm to 15 µm in length^5,6,18,19^ and when in bulk appear as a black powder^20^. Respirable particles have the capacity to deposit in the alveolar region of the lungs creating great concern for human toxicity. The physical characteristics of CNTs give them the ability to elicit pathological responses similar to asbestos, therefore, accurate characterization of worker exposure is imperative. Agglomerates of CNTs larger than the respirable size range have previously been identified in workplaces^21,22^ using the sampling methods used by Dahm *et al*. a research industrial hygienist at the U.S. National Institute for Occupational Safety and Health (NIOSH). There is suspicion that the multitude of large agglomerates and rarity of single fibers previously identified is due to the type of matrix the CNT substrate exists in^22^. Some other studies have also used similar methods to sample and characterize CNTs in workplaces^23–27^. However, limited research of CNT release from a bulk powder has been conducted to confirm or deny this hypothesis. The fiber sampling and analytical method must be evaluated to determine if it discriminates against individual small fibers so that accurate exposure assessments can be conducted.

The currently employed fiber analytical method in the U.S., NIOSH Manual of Analytical Methods (NMAM) 7402, has been modified for CNT characterization, and relies on a solvent-based transfer process^28^. Although NMAM 7402 works well on most asbestos fibers in the micrometer size ranges, a NIOSH study in 1990 has documented the loss of small fibers trapped inside the mixed cellulose ester (MCE) filter or washed away during the transfer process^29^. Another concern with NMAM 7402 has been the possible uneven distribution of asbestos structures across the filter because only a small portion of the specimen gets analyzed^30^. This method can also lead to an underestimation of the presence of asbestos structures because other particles may obscure some asbestos fibers^31^. Using a MCE filter as instructed in NMAM 7402 to collect CNTs with fibers that are much smaller than asbestos requires further investigation regarding the efficiency and possible alteration of fiber morphology through the sampling and sample analysis. It has been hypothesized that the transfer process required by NMAM 7402 may have an impact on the observed CNT fiber morphology and size distribution. This case study sought to characterize the morphology and size distribution of CNTs collected by two methods; (1) the 25 mm open-faced cassette following NMAM 7402 modified for CNT sampling and (2) a novel direct sampling and analysis method - a newly developed sampler named the Tsai Diffusion Sampler (TDS)^32^. The performances of two studied methods were evaluated for this case study. The goal of this study was to identify possible differences contributed by different sampling and analytical methods to improve the assessment for CNT exposure.

## Results and Discussion

This section includes analysis of CNT particles and clusters collected and/or prepared on filters and/or grids. The estimated concentrations and sizing analysis of particles on transmission electron microscope (TEM) images are presented first, followed by the analysis of particles deposited on filters using scanning electron microscope (SEM). Then, we present the real time instrument measurements to confirm the findings of particles collected and analyzed using studied two methods. At the end, we determine the impact of using different methods to characterize CNT fibers.

### TEM analysis for particle counting and concentration extrapolation

**Figure 1:**
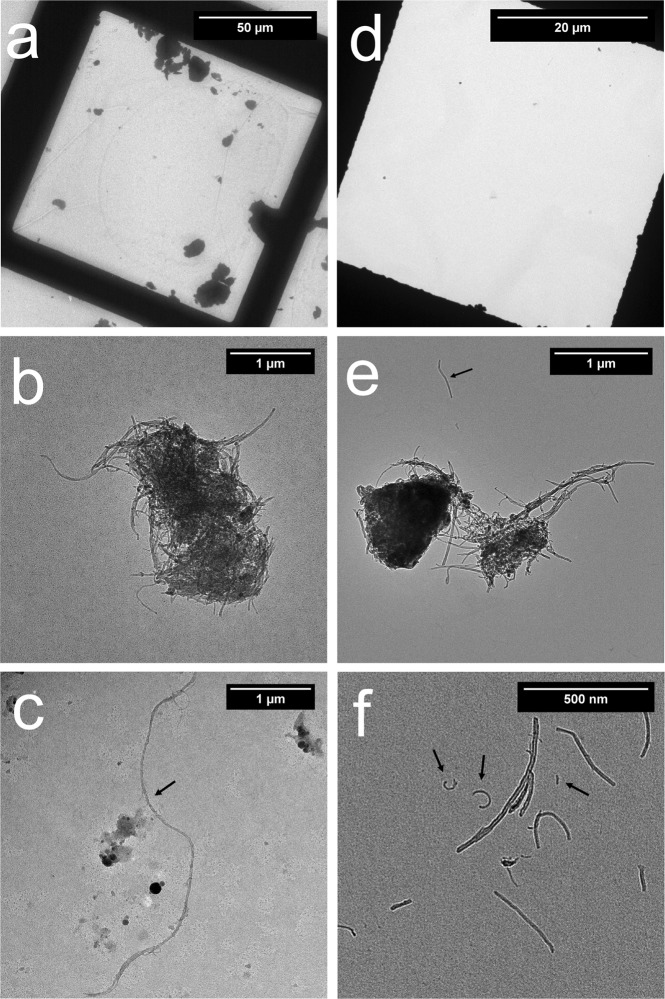
TEM images of sampled CNT fibers and agglomerates on grid. (**a**–**c**) Are samples collected using the 25 mm open-faced cassette following NMAM 7402, (**a**) overview of a grid opening area, (**b**) an agglomerate, (**c**) a single fiber; (**d**–**f**) are samples collected by the TDS, (**d**) overview of a grid opening area, (**e**) agglomerates, (**f**) single fibers. Arrow marks note for single or small fibers. The scale bar for (**a**) is 50 μm, (**b**) is 1 μm, (**c**) is 1 μm, (**d**) is 20 μm, (**e**) is 1 μm, and (**f**) is 500 nm.

**Figure 2:**
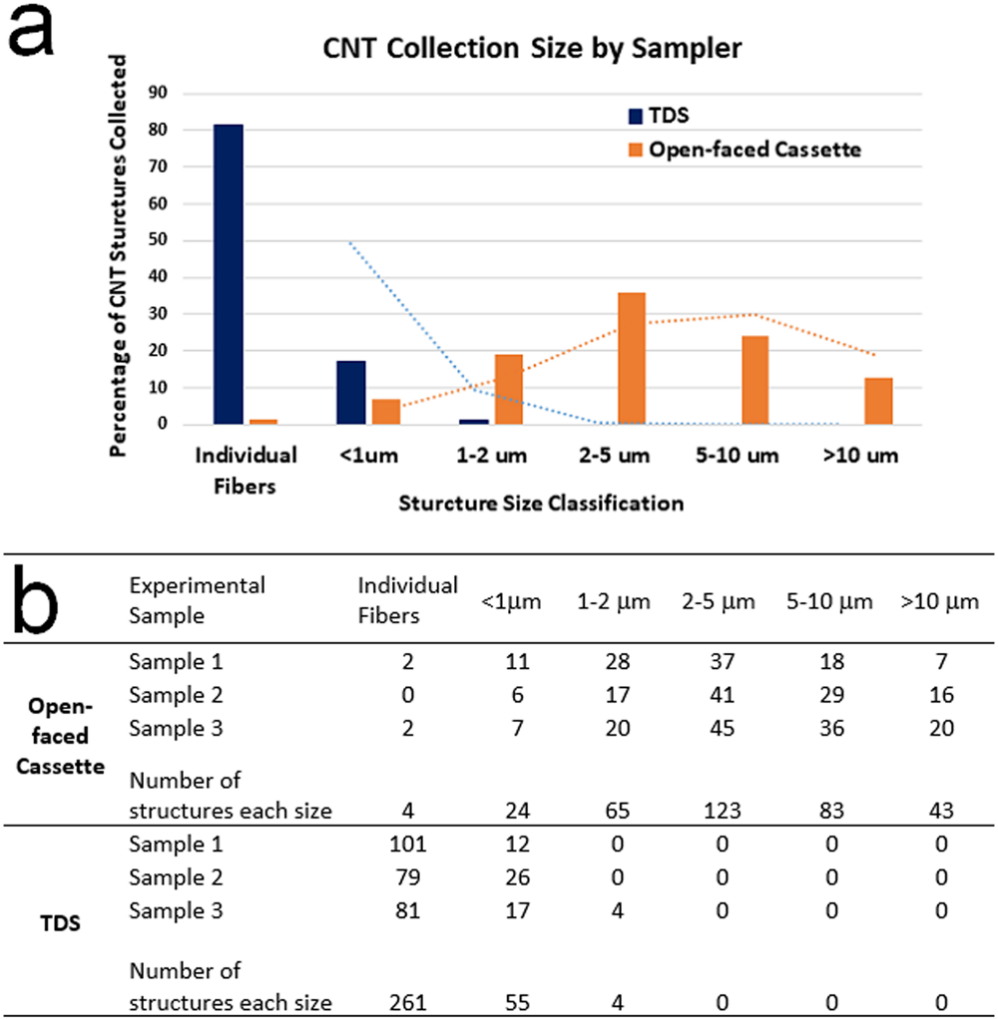
(**a**) Percentage and (**b**) counts of fiber structures from TEM images at each size category.

The quantitative analysis including particle sizing and counting is presented based on data analyzed from TEM images taken on each grid sample. Concentration estimates for both sampling methods are calculated in accordance with NMAM 7402. Examples of images are shown in Fig. 1a–c for CNT fibers/clusters using the 7402 method and Fig. 1d–f for CNTs using the TDS method. These example images show an overview of a grid opening (Fig. 1a,d), agglomerates/clusters (Fig. 1b,e) and individual fibers (Fig. 1c,f). The researchers counted an agglomerate, or cluster, with obvious bonding as one structure as seen in Fig. 1b for 1 structure and 1e for 2 structures. Due to the size of the small fibers, they can’t be observed on the overview images (Fig. 1a,d). The researchers counted the total number of CNT structures (individual fiber and agglomerate) from images taken on grids from three repeated samples. Using a 25 mm open-faced cassette prescribed in the 7402 method, of the total fibers counted, 342 structures/fibers were counted on 12 grid openings from three repeated experiments/grids on the samples. The samples from the first, second, and third experiments required 4, 6, and 2 grid openings respectively to attain 100 or more counted structures as required by the asbestos fiber counting rule. As presented in Fig. 2a with data listed in Fig. 2b, the researchers observed 1 percent of total structure count as individual fibers, 7 percent as clusters less than one micrometer (μm) in size (maximum crosswise dimension), 19 percent as clusters one to two micrometer in size, 36 percent as clusters two to five micrometer in size, 24 percent as clusters five to ten micrometer in size, and 13 percent as clusters larger than ten micrometer in size.

The analysis of the grid samples collected by the TDS includes the counts from a combined total of 320 particles on four grid openings from three experiments. Samples from experiment one and two only required particles from one grid opening to be counted to attain 100 or more counted particles; the sample from the third experiment required particles from two grid openings to be counted to attain a minimum of 100 counted structures. As presented in Fig. 2a,b, of the total 320 fiber structures counted from 3 grids (4 grid openings) of TDS samples, 82 percent were individual CNT fibers, 17 percent were clusters/agglomerates less than one micrometer in size, and one percent were clusters one to two micrometer in size. No fibers or clusters larger than two micrometer in size were observed on the TEM grids of TDS samples. CNT fibers collected using a 25 mm open-faced cassette and following the NMAM 7402 were mostly in the micrometer size range as seen in Fig. 2, while the fibers collected on the grid by the TDS were mostly in the smaller, sub-micrometer size (Fig. 1f). CNT clusters collected by the TDS on the polycarbonate filter which the grid is attached on are presented in the following section. In addition, particles not consistent with CNT aerosol morphology were often found on grid samples prepared using the 7402 method. These non-fibrous particles surrounding the arrow marked CNT fiber as seen in Fig. 1c may have been created during the transfer process.

The significant discrepancy in the number of individual and sub-micrometer fibers/structures collected by each sampler (Fig. 2) can be explained by differences in the sampler design. Important differences include filter type, collected particle sizes, operating conditions (e.g., air flow), and required sample preparation steps. The TDS collects particles with an aerodynamic cutoff diameter, at a size of 50% cumulative concentration (D_50_) of approximately 4 µm, which is considered a respirable size. This is determined by the inlet diameter and low air flow designed for this use^32^. The sampler used in this study following the NMAM 7402 is an open-faced three piece cassette^22,33^ which does not discriminate against large agglomerates to the extent of the TDS. This allows for the collection of all sizes of fiber bundles in the air that include the inhalable size range. The TDS collects particles directly onto a grid and filter with a low air flow (0.3 L/min), which enhances diffusion deposition of smaller particles. Conversely, the 25 mm open-faced cassette following 7402 method operates at a much higher air flow of 4 L/min. The 7402 method requires a wedge of the MCE filter with deposited CNT particles to be transferred onto a TEM grid using dimethylformamide (DMF) as a solvent to dissolve the MCE filter following a carbon coating process^28^. The transfer and dissolution process might have an effect on particle morphology or result in the loss of individual fibers. In addition, the different deposition mechanisms primarily employed by each sampler, diffusion and impaction, may have caused the contrast seen in particle sizes collected.

Based upon the number of CNT structures counted in the specific size bins selected in this study (shown in Fig. 2b), an airborne fiber/particle concentration estimate was calculated using Equation (1). The estimated average total particle number concentrations are 5,200 (±2,100) fiber structures/cm^3^ and 59 (±9) fiber structures/cm^3^ for TDS sampler and 25 mm open-faced cassette following NMAM 7402 respectively. As shown in Equation (1), concentration estimates are calculated by dividing the volume of air sampled and the number of grid openings counted. As these variables increase, the estimated concentration decreases. However, these two samplers were operated side by side to characterize the same CNT aerosol.

### Filter characterization by SEM

**Figure 3:**
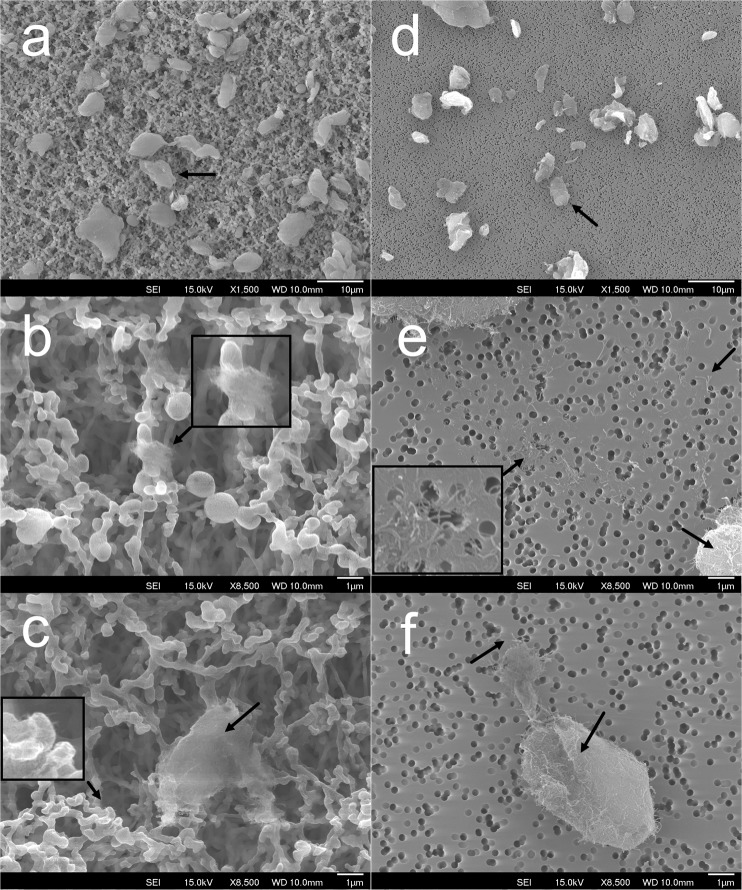
Examples of filter sample taken by SEM. (**a**–**c**) Images of the CNT structures collected on a MCE filter by the 25 mm open-faced cassette; (**d**–**f**) Images of CNT structures collected on a polycarbonate filter by the TDS. Arrow marks note examples of CNT structure and fiber on filter. The scale bar for (**a**) is 10 μm, (**b**) is 1 μm, (**c**) is 1 μm, (**d**) is 10 μm, (**e**) is 1 μm, and (**f**) is 1 μm.

The sampled polycarbonate filters used by the TDS sampler and MCE filters used by the 25 mm open-faced cassette following NMAM 7402 were analyzed using SEM to characterize the collected CNT fibers and filter structures. Examples of images taken by the SEM are presented in Fig. 3a–f. Large CNT agglomerates were seen on both filters (Fig. 3a,d). The MCE filter which has approximately 450 nm sized pores on the fibrous structured filter is seen in Fig. 3b,c. The polycarbonate membrane filter as seen in Fig. 3e,f has pores that are approximately 200 nm in diameter. CNT agglomerates were seen trapped on fibrous structure of the MCE filter (Fig. 3b,c) with a few individual/small fibers observed as marked at the left bottom of Fig. 3c. Identification of such small fibers on an MCE filter by SEM was difficult due to the filter structure not being suitable for microscopic analysis. Numerous small and individual fibers were observed between the large agglomerates on the polycarbonate filter as seen in Fig. 3e. Agglomerates with similar size in micrometers as to those found on the MCE filter were also observed on the polycarbonate filter (Fig. 3f).

It was observed in Fig. 3a,d that the MCE filter had more CNT structures than the polycarbonate membrane filter from the same sampling period of CNT collection. This result was expected as this sampler uses an air flow (4 L/min) that is about 13 times higher than the TDS (0.3 L/min) and operates with an open-faced cassette for collecting particles. The majority of CNT structures found on the grid sample from the 25 mm open-faced cassette following 7402 method exceeded 2 micrometer in size with many being in excess of 5 and 10 micrometer. The estimated fiber number concentration calculated with all parameters (Equation (1)) was much lower on the samples by the 25 mm open-faced cassette following 7402 method than the TDS samples, and a few individual fibers were identified on the grid sample prepared from the MCE filters. However, these individual fibers represent that majority of the CNT structures collected on the grid of the TDS for all samples collected. As discussed in the previous section, the lack of individual fibers on the grid sample transferring from the MCE filters collected by the 25 mm open-faced cassette were related to the sampling flowrate, porosity of the filter, and the fiber transfer from filter to grid. As seen in Fig. 3b,c, small CNT clusters containing single fibers were trapped on the fibrous structure of MCE filter fiber and the porous structure would allow small CNT fibers to flow into the filter. Single fibers may imbed into the filter where carbon coating is rendered ineffective in locking the fibers in place for transfer to TEM grid.

### Direct reading real time instrument measurements

**Figure 4:**
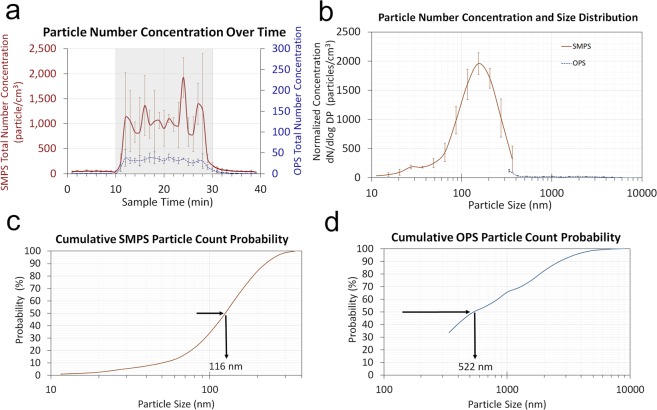
Airborne particle/fiber concentrations and sizes measured by direct reading instruments, (**a**) Average total particle number concentrations during 40 min experiment with nanoscan SMPS data (10–420 nm) referring to the left y-axis and OPS data (300–10000 nm) referring to the right y-axis, (**b**) Particle average concentrations and size distributions during 15 min sampling period, (**c**) Cumulative particle count probability of nanoscan SMPS data, (**d**) Cumulative particle count probability of OPS data.

Airborne CNT fiber concentrations measured by the direct reading instruments (DRIs) nanoscan SMPS and OPS throughout the entire experiment were found to be relatively consistent during the sampling period as seen in Fig. 4a. The average total number concentration measured by the nanoscan SMPS and the OPS during CNT generation was 1,100 (±25) particles/cm^3^ (10–420 nm) and 33 (±4) particles/cm^3^ (0.3–10 μm) respectively (Fig. 4a). Based upon the size distribution graph shown in Fig. 4b, the majority of CNT structures generated were less than 200–300 nm in size. The relative D_50_ measured by the nanoscan SMPS and OPS were 116 nm and 522 nm respectively (Fig. 4c,d).

The DRIs measured the aerosol generated to be primarily in the sub-micrometer (<500 nm) range. However, when compare the DRI measurements to the CNT structure estimates shown in Fig. 2, the concentration of sub-micrometer particles observed by the DRIs was high compared to the few on the SEM imaged MCE filter and *none* on the TEM imaged grid from the 25 mm open-faced cassette following the modified 7402 method. This sub-micrometer fraction of the aerosol was found in abundance on the samples collected by the TDS on both the polycarbonate membrane filter and the TEM grid. The results presented in this case study also indicate that sampling airborne fibers such as asbestos using NMAM 7402 in the past could have been underestimating the ultrafine fibers and fail to present representative results if such small fibers exist in the air. Characterization and quantitative measurements on exposed CNT fibers need to be as comprehensive as possible. Analysis of CNTs sampled directly onto an imaging substrate ensures the integrity of the sample leaving little room for alteration of collected particles. The small and individual CNT fibers need to be properly quantified to perform through exposure assessments and health effect evaluations. It is relevant to the sampler design, substrate use, and operating procedure that different size of particles are collected by each sampler. The use of both samplers is warranted to ensure accurate characterization of a full range including micron and submicron CNT structures.

## Methods

### Process and equipment

To demonstrate the practical exposure from manual activities, airborne CNT fibers were generated through manual stirring inside of an enclosure placed inside of a glovebox (89 cm × 61 cm × 64 cm) equipped with ultra-filter (manufactured by Terra Universal, Fullerton, CA, USA). The multi-walled CNTs (research grade, >95% purity, approximately 12 nm diameter) were purchased from Nanolab Inc. (Waltham, MA, USA). The air velocity measured at the sampling location was 0.01 to 0.07 m/s in the horizontal direction and 0.00 to 0.03 m/s in the vertical direction. Airborne particle concentrations were measured using DRIs. Airborne CNT fibers were collected using (1) open-faced 25 mm three piece sampling cassette and following the modified 7402 method^28^ and (2) TDS sampler^32^.

The TDS sampler, patent pending, is designed to collect respirable particles containing nanoparticles/ultrafine particles; its design gives novel features unavailable from other respirable particle samplers. The novel design aspects of this sampler include the selection of the diameter of the inlet probe, geometry of the sampler, and the resulting air flow to the sampler^32^. The cutoff diameter, which was determined experimentally to be a mass median aerodynamic diameter (MMAD) of 3.8 µm^32^. The low air flow (0.3 L/min) sampling operated in TDS enhances the collection of nanoparticle/ultrafine particle as a result of strong Brownian motion and laminar airflow entering cassette and flowing through the polycarbonate filter constructed with pores as small as 0.22 μm diameter. TDS collects particles on a substrate consisting of a TEM grid attached to a polycarbonate membrane filter and particles are collected on both the TEM grid and filter. The theoretical diffusion efficiency for collecting particles on this polycarbonate filter is 100% for particles with diameter of 0.5 μm or smaller and greater than 95% for particles of 1–0.5 μm^32^. Particles collected on grids are analyzed directly by TEM without a transfer or treatment procedure. Particles collected on the polycarbonate membrane filter are directly analyzed using SEM without a transfer procedure.

This evaluation was based upon the CNTs collected on the polycarbonate filter and TEM grid by TDS and on the MCE filter then transferred to a TEM grid from the 25 mm open-faced cassette and following NMAM 7402 to perform analysis. Each experiment lasted forty minutes and stirring was conducted for fifteen minutes; three repeated experiments were performed. The DRIs used for this case study include a nanoscan scanning mobility particle spectrometer (nanoscan SMPS) (Model 3910, TSI, Shoreview, MN, USA), which measures particle sizes of 10 to 420 nm in 13 size channels and the optical particle sizer (OPS) (Model 3330, TSI, Shoreview, MN, USA) which measures particle sizes of 0.3 to 10 μm in 16 size channels. The nanoscan SMPS uses an air flow of 0.9 L/min and the OPS has an air flow of 1.0 L/min. The maximum concentrations that can accurately be measured by the nanoscan SMPS and OPS are 1,000,000 particles/cm^3^ and 3,000 particles/cm^3^, respectively. The nanoscan SMPS, OPS, TDS, and 7402 sampler were operated side by side equidistant to the beaker.

Samples were taken directly adjacent to aerosol generation approximately 5 cm from the beaker top opening. The TDS, sampling at 0.3 L/min, was placed inside the enclosure equidistant to the beaker and away from the 25 mm open-faced cassette (4 L/min) because of its significantly lower flow rate. Samples were only taken during the stirring period (15 min), while the DRIs measured concentrations during the whole experimental period. The TDS^32^ collects small CNT fibers directly onto a TEM-copper grid in 400 mesh with SiO_2_ coated film (SPI, West Chester, PA, USA) which is attached to the center of the 0.22 µm pore polycarbonate filter (Isopure, Millipore Sigma, Burlington, MA, USA); this allows for nanoparticles and respirable particles to be collected simultaneously on the grid and filter. Respirable sized CNT fibers and their agglomerates are mostly collected on the polycarbonate filter with sub-micrometer (<500 nm) or nanometer sized fibers collected on the grid^32^. The sampler used in this study following the analytical modified 7402 method uses a three piece, 25 mm open faced cassette^21^; it collects particles onto a 25 mm diameter, 0.45 µm porosity MCE filter, which then requires particles to be transferred onto an un-filmed TEM grid. In this study, CNT fibers collected on both filters and grids from each sampler were analyzed.

### Evaluation of carbon nanotube collection

CNT fiber counts and size distributions from each sampler were evaluated using microscopic analysis. MCE filter samples collected using the modified 7402 method were sent to a certified vendor to process and transfer fibers from the filter (a portion) to the grid. The prepared grid samples and MCE filters were returned, and the grids and polycarbonate filters were removed from the TDS samplers and directly analyzed. All samples were analyzed using the same microscope facility.

TEM grids were imaged using a JEOL JEM2100F TEM at 200 kV. Grid spaces were checked first at low magnification for general distribution and uniformity of collected fibers. A grid space close to the center was chosen first and an image of an entire grid space was taken. Next, images of every CNT fiber and agglomerate or cluster (as called structure) in that grid space were taken. Images of CNTs were then taken on grid spaces adjacent to the first one until 100 or more structures were found. The grid samples by 25 mm open-faced cassette following NMAM 7402 were initially surveyed at higher magnifications as used for analysis of the TDS samples in a failed attempt to identify single fibers that may not be visible due to the magnification constraints prescribed in the 7402 method. Thus, a magnification of 400x to 6,000x was used for the TEM grid samples which were prepared in accordance with the modified 7402 method^28^. TDS TEM samples were examined at a magnification of 5,000x to 15,000x. This magnification range was used based on its ability to observe CNT agglomerates and fibers which were much smaller than those observed on samples collected using 25 mm open-faced cassette with MCE filter and following modified NMAM 7402. For both samples, CNT structures were counted according to the modified 7402 method for CNT collection and analysis. The researchers counted all individual fibers with a greater than 3:1 aspect ratio; clusters were characterized by their maximum crosswise dimension and then placed into size bins of <1 µm, 1–2 µm, 2–5 µm, 5–10 µm, and >10 µm. Gatan digital micrograph allowed the researchers to determine the crosswise dimension of each CNT cluster/agglomerate.

The researchers imaged the CNTs collected on the polycarbonate membrane filters and MCE filters for each sample using a JEOL JSM-6500F SEM at 15 kV operating voltage. A wedge shape filter piece from each sample was cut and sputter coated with 20 nm of gold for SEM analysis. The researcher took 10 images of each filter piece at 1,500x evenly from the edge to the center of the filter.

### Estimation of airborne fiber concentration

Utilization of Equation (1) allows the researchers to estimate airborne CNT concentrations from fiber counting on the TEM grids. This equation is typically used to calculate the estimated airborne fiber concentrations for asbestos characterization. For calculating the concentrations of fibers collected by the TDS, the equation was modified for the TDS filter and grid size. The TDS has an effective filter area of 415 mm^2^ and a grid opening area of 1.37 × 10^3^ mm^2^. The samples from the MCE filter following the modified 7402 method have an effective area of 385 mm^2^ and a grid opening area of 0.01 mm^2^.

1 $$\begin{array}{ccc}\frac{Number\,of\,CNT\,Structures}{c{m}^{3}} & = & \frac{{\rm{Number}}\,{\rm{of}}\,CNT\,structures}{{\rm{Number}}\,{\rm{of}}\,Grid\,Openings\,Counted}\\  &  & \times \,\frac{1}{Volume(L)}\times \,\frac{Effective\,Filter\,Area\,(m{m}^{2})}{Average\,Grid\,Opening\,Area\,(m{m}^{2})}\times \frac{1L}{1000cc}\end{array}$$

Estimation of airborne number concentration from fiber image counting.
